# Creatine Kinase-Overexpression Improves Myocardial Energetics, Contractile Dysfunction and Survival in Murine Doxorubicin Cardiotoxicity

**DOI:** 10.1371/journal.pone.0074675

**Published:** 2013-10-01

**Authors:** Ashish Gupta, Cory Rohlfsen, Michelle K. Leppo, Vadappuram P. Chacko, Yibin Wang, Charles Steenbergen, Robert G. Weiss

**Affiliations:** 1 Department of Medicine, Division of Cardiology, the Johns Hopkins University School of Medicine, Baltimore, Maryland, United States of America; 2 Department of Radiology, Division of Magnetic Resonance Research, the Johns Hopkins University School of Medicine, Baltimore, Maryland, United States of America; 3 University of California Los Angeles, Los Angeles, California, United States of America; 4 Department of Pathology, the Johns Hopkins University School of Medicine, Baltimore, Maryland, United States of America; University of Washington School of Medicine, United States of America

## Abstract

Doxorubicin (DOX) is a commonly used life-saving antineoplastic agent that also causes dose-dependent cardiotoxicity. Because ATP is absolutely required to sustain normal cardiac contractile function and because impaired ATP synthesis through creatine kinase (CK), the primary myocardial energy reserve reaction, may contribute to contractile dysfunction in heart failure, we hypothesized that impaired CK energy metabolism contributes to DOX-induced cardiotoxicity. We therefore overexpressed the myofibrillar isoform of CK (CK-M) in the heart and determined the energetic, contractile and survival effects of CK-M following weekly DOX (5mg/kg) administration using *in vivo* ^31^P MRS and ^1^H MRI. In control animals, *in vivo* cardiac energetics were reduced at 7 weeks of DOX protocol and this was followed by a mild but significant reduction in left ventricular ejection fraction (EF) at 8 weeks of DOX, as compared to baseline. At baseline, CK-M overexpression (CK-M-OE) increased rates of ATP synthesis through cardiac CK (CK flux) but did not affect contractile function. Following DOX however, CK-M-OE hearts had better preservation of creatine phosphate and higher CK flux and higher EF as compared to control DOX hearts. Survival after DOX administration was significantly better in CK-M-OE than in control animals (*p*<0.02). Thus CK-M-OE attenuates the early decline in myocardial high-energy phosphates and contractile function caused by chronic DOX administration and increases survival. These findings suggest that CK impairment plays an energetic and functional role in this DOX-cardiotoxicity model and suggests that metabolic strategies, particularly those targeting CK, offer an appealing new strategy for limiting DOX-associated cardiotoxicity.

## Introduction

Doxorubicin (DOX) is an antineoplastic agent often used for treatment of advanced solid tumors and several hematopoietic malignancies [1,2]. However, DOX’s therapeutic application is limited in part by dose-dependent adverse effects on cardiac function [3,4], resulting in heart failure (HF) [5-7]. A 250-601 mg/m^2^ cumulative dose of DOX has been reported to cause abnormalities in systolic and diastolic function in 18-36% of patients [3].

DOX treatment causes several myocardial metabolic and morphologic changes [8,9] and a number of molecular mechanisms have been hypothesized to contribute to its cardiotoxicity [10-13]. Although DOX-induced cardiotoxicity may be a multi-factorial process, it seems that impaired energy metabolism may be a critical, common factor [1,2,14-17]. ATP is absolutely required for normal cardiac contractile function and DOX reduces the cardiac high-energy phosphates, ATP and creatine phosphate (PCr), as well as the myocardial PCr/ATP ratio in experimental models and in people [18-22]. DOX also inhibits the creatine kinase (CK) reaction, the prime myocardial energy reserve reaction which rapidly and reversibly exchanges a phosphoryl group between ATP and PCr [23-26]. Following chronic DOX administration in a murine model, myocardial energetic changes, indexed by a reduced PCr/ATP ratio, occur relatively early and precede the appearance of contractile dysfunction and predict the subsequent degree of contractile abnormalities [27], suggesting, but not proving, a causal role for impaired energy metabolism in subsequent DOX-induced cardiac dysfunction.

However it is not clear whether the decline in CK metabolism contributes to DOX-induced cardiac dysfunction or is simply another consequence of DOX. To determine the role of impaired CK metabolism in DOX-induced cardiotoxicity, we studied the consequences of cardiac-specific over-expression of the myofibrillar isoform of CK (CK-M-OE), the predominant CK isoform in the adult heart, in mice administered DOX. We recently reported that CK-M-OE does not alter contractile function in the normal heart but that in mice with HF induced by thoracic aortic constriction (TAC), CK-M-OE improves impaired energetics, contractile function at rest and during adrenergic stress, and increases survival [28,29]. We therefore exploited these CK-M-OE mice to address the role of impaired CK metabolism in DOX cardiotoxicity by exposing them and non-transgenic littermates to a DOX regimen previously shown to induce contractile dysfunction [27]. We used non-invasive ^31^P magnetic resonance spectroscopy (MRS) to determine the effect of CK-M-OE on *in vivo* cardiac energetics (PCr and ATP levels and the rate of ATP synthesis through CK) and the subsequent impact on left ventricular function and remodeling in hearts chronically administered DOX. We also determined the effect of CK-M overexpression on survival. These integrated studies were designed to test the hypothesis that CK-M-OE improves impaired cardiac energetics, contractile dysfunction, and survival in murine DOX-induced cardiotoxicity.

## Methods

### Animal Studies

The procedure and protocols were reviewed and approved by the Institutional Animal Care and Use Committee of the Johns Hopkins University (#MO10M139). All transgenic mice development work was performed at the transgenic core facility of the University of California, Los Angeles (UCLA), USA. Transgenic mice (CK-M-OE) were developed on the basis of the Tet-off system [30]. At the first stage, two types of mice were generated; first, those with a transgene for the regulatory protein tTA (tetracycline-controlled transactivator) under the control of α-MHC promoter and second, mice expressing the CK-M transgene under the control of a tetracycline-responsive element (TRE) following microinjection of the CK-M construct into fertilized mouse embryos (C57BL/6 strain) [28]. After the crossing of TRE-CK-M mice with α-MHCtTA mice, double transgenic mice (CK-M-tTA) were confirmed by genotyping and CK-M transgene induction was achieved by switching from a diet containing doxycycline (650 mg/kg body weight) to a regular chow diet at least four weeks prior to study (hereafter called CK-M-OE). Control mice were littermates containing either the tTA or CK-M transgenes alone or double transgenic CK-M-tTA mice with the CK-M transgene turned “off” by maintenance of the doxycycline diet since these three groups have similar, normal myocardial CK activity [28,29].

CK-M-OE and control adult littermate mice were administered intra-peritoneal DOX (5mg/kg) once a week for five weeks. The DOX dosing schedule was adapted from that previously reported [27]. The cumulative DOX dose was 25mg/kg (or ~180mg/m^2^). To determine the effect of DOX administration on lung/body weight ratios, other control and CK-M-OE animals were sacrificed after five weekly injections of DOX or an equal volume of saline.

### In vivo MRI/MRS studies

All *in vivo* MRI/MRS experiments were carried out on a Bruker Biospec MRI/MRS spectrometer equipped with a 4.7T/40cm Oxford magnet and a 12cm (inner diameter) actively shielded gradient set, as previously described [28-33]. Specifically, mice were positioned on a Plexiglas platform housing 22-mm ^1^H MRI and 13-mm ^31^P MRS coils and with temperature control (37±1°C). Anesthesia was induced with ~2% isoflurane in an induction chamber and maintained at ~1% in 50/50 air/oxygen mixture delivered through a nose cone, as previously described [28,32,33]. Electrocardiographic (ECG) and respiratory monitoring were performed continuously during MRI/MRS experiments. Because some chronically ill DOX-administered mice do not tolerate prolonged anesthesia, the functional MRI and energetic MRS studies were performed separately. Cine MRI experiments were performed at 6 and 8 weeks after DOX administration for left ventricular (LV) function and morphology in a fashion comparable to an earlier, separate study [27]. Multi-slice FLASH cine MR images were acquired (15 frames, TE = 1.5 ms, TR = 7ms, flip angle = 45°) of the entire LV to assess LV mass, ventricular volumes and ejection fraction (EF) [28,32,33]. MRIs were analyzed with ImageJ software. The largest and smallest LV volumes were visually identified as end-diastolic (EDV) and end-systolic volume (ESV). LV mass was the sum of all end-diastolic cross-sectional slices of the LV epicardial and endocardial area difference multiplied slice thickness and by 1.05 (cardiac tissue-specific gravity). The LV EF was calculated from the relative difference in EDV and ESV. A separate ^31^P MRS protocol including the Triple Repetition Time Saturation Transfer (*TRiST*) method for ATP kinetics was used during the 7^th^ week of DOX administration to measure the rate of ATP synthesis through CK (CK flux) from the product of [PCr] and (*k*_*f*_), the CK pseudo-first order rate constant, as previously described [29,31]. Specifically, localized ^31^P MR spectra were obtained with a one-dimensional chemical shift imaging sequence (16 mm field of view, 16 phase encoding steps) using modified BIR4 90° adiabatic pulses. Two acquisitions with different repetition periods (TR=1.5s, NEX=96 and TR=6s, NEX=32) were acquired in the presence of a saturating irradiation pulse applied to the exchanging CK moiety, *viz*., γ-phosphate of ATP at -2.5 ppm, relative to PCr, and another acquisition, fully relaxed (TR=10s, NEX=16) in the presence of control irradiation applied at +2.5ppm relative to PCr. The measured signal intensities were normalized for different NEX. Absolute [PCr] and [ATP] were evaluated *in vivo* as described previously [31,34]. Animals that were sacrificed were given isoflurane anesthesia until a deep level of anesthesia was obtained, as documented per toe pinch, and then the hearts were removed.

To determine whether CK-M overexpression improved survival during DOX administration, a separate study was performed with CK-M-OE (n=32) and control (n=39) mice which were followed for 8 weeks after DOX administration (intra-peritoneal DOX 5mg/kg weekly for 5 weeks for a cumulative dose of 25mg/kg) with daily cage census. Eight mice (five controls and three CK-M-OE) were euthanized for humane reasons once it became clear the mice were terminally ill.

## Statistical Analyses

Results are presented as mean ± standard deviation (SD). Survival curves were generated using the Kaplan-Meier survival function using statistical XLSTAT. Comparisons of MRI- and MRS-derived measures of LV anatomy, function and metabolism among multiple groups were analyzed by one way ANOVA and significant differences were assessed with the post hoc Student-Newman-Keuls multiple comparisons test.

## Results

**Figure 1:**
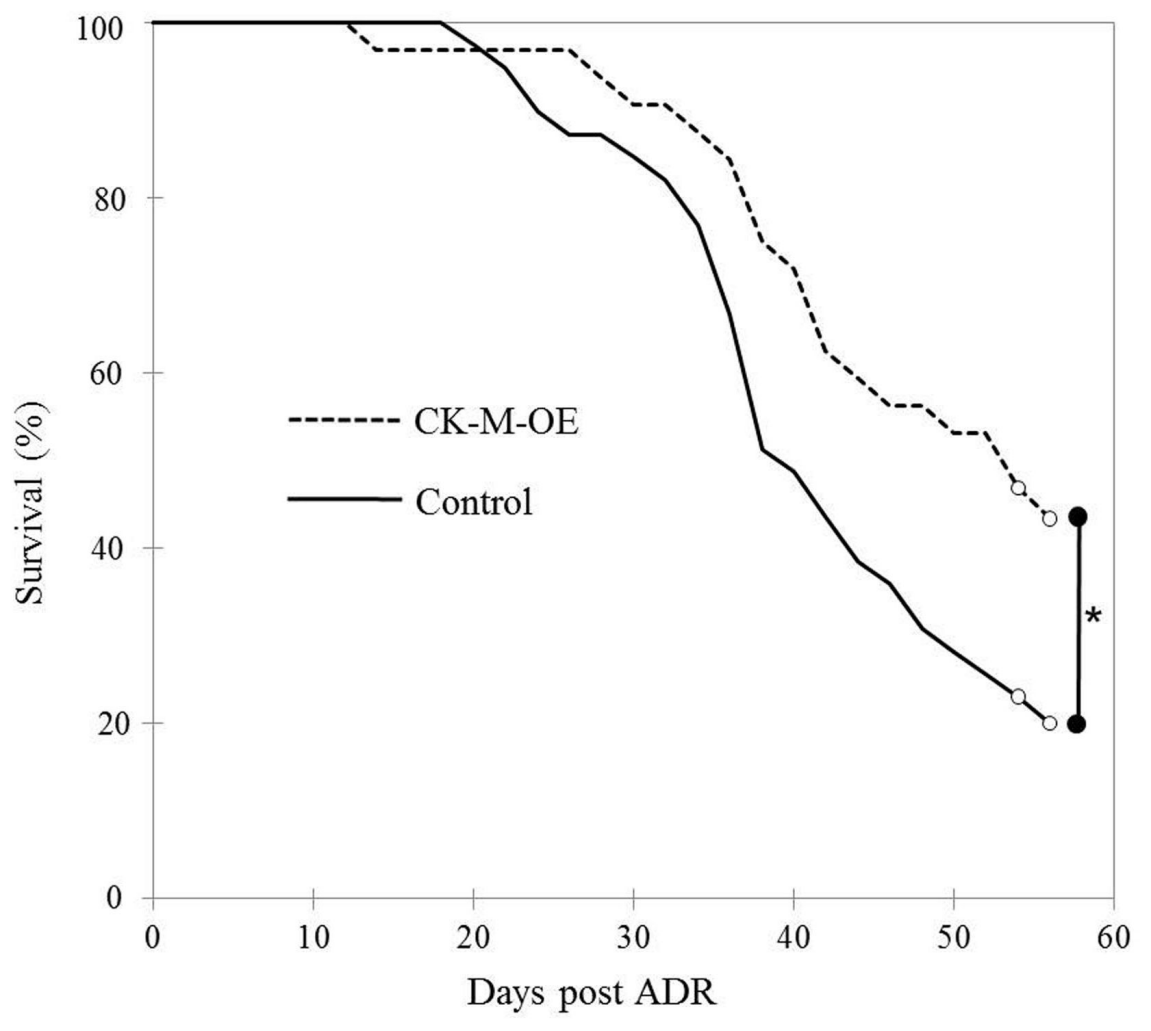
CK-M overexpression improves survival following DOX administration in mice. Kaplan-Meier survival curves showing higher survival following DOX in CK-M-OE mice (dotted line *n* = 32) as compared with control mice (solid line, *n* = 39). *P <0.02,.

At baseline, prior to DOX, control and CK-M-OE mice had similar body weights (40.0±6.4g vs 42.2±8.4g, respectively, *p*= NS). Mice lost weight during DOX administration, as previously observed, but the mean weight loss was similar in control and CK-M-OE animals (-12.3±6.0g vs -11.1±6.1g, respectively, *p* = NS). Importantly, the mortality after 8 weeks of DOX administration was ~80% in control mice but only ~56% in CK-M-OE mice (*p*<0.02, Figure 1).

**Figure 2:**
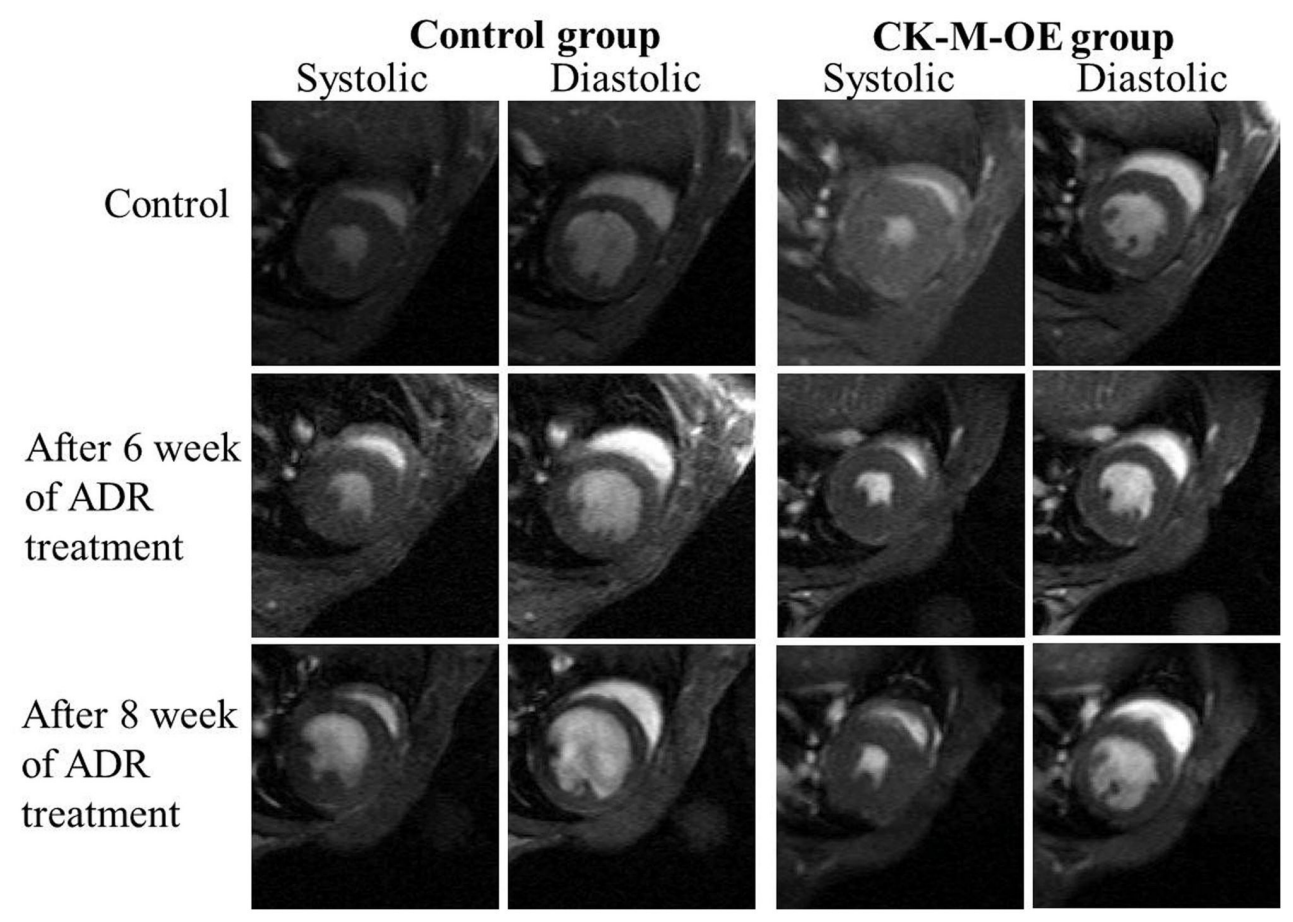
In vivo MRI reveals murine cardiac morphologic and functional changes after DOX. Typical transverse short-axis *in* *vivo* ^1^H MR images of the mid-left ventricle at end-systole and end-diastole at baseline (top row), 6 weeks (middle row) and 8 weeks (bottom row) in control (left panel) and CK-M-OE (right panel) mice. LV= left ventricle, RV= right ventricle, P= spherical fiducial phantom embedded in MR surface coil outside of the mouse body.

**Figure 3:**
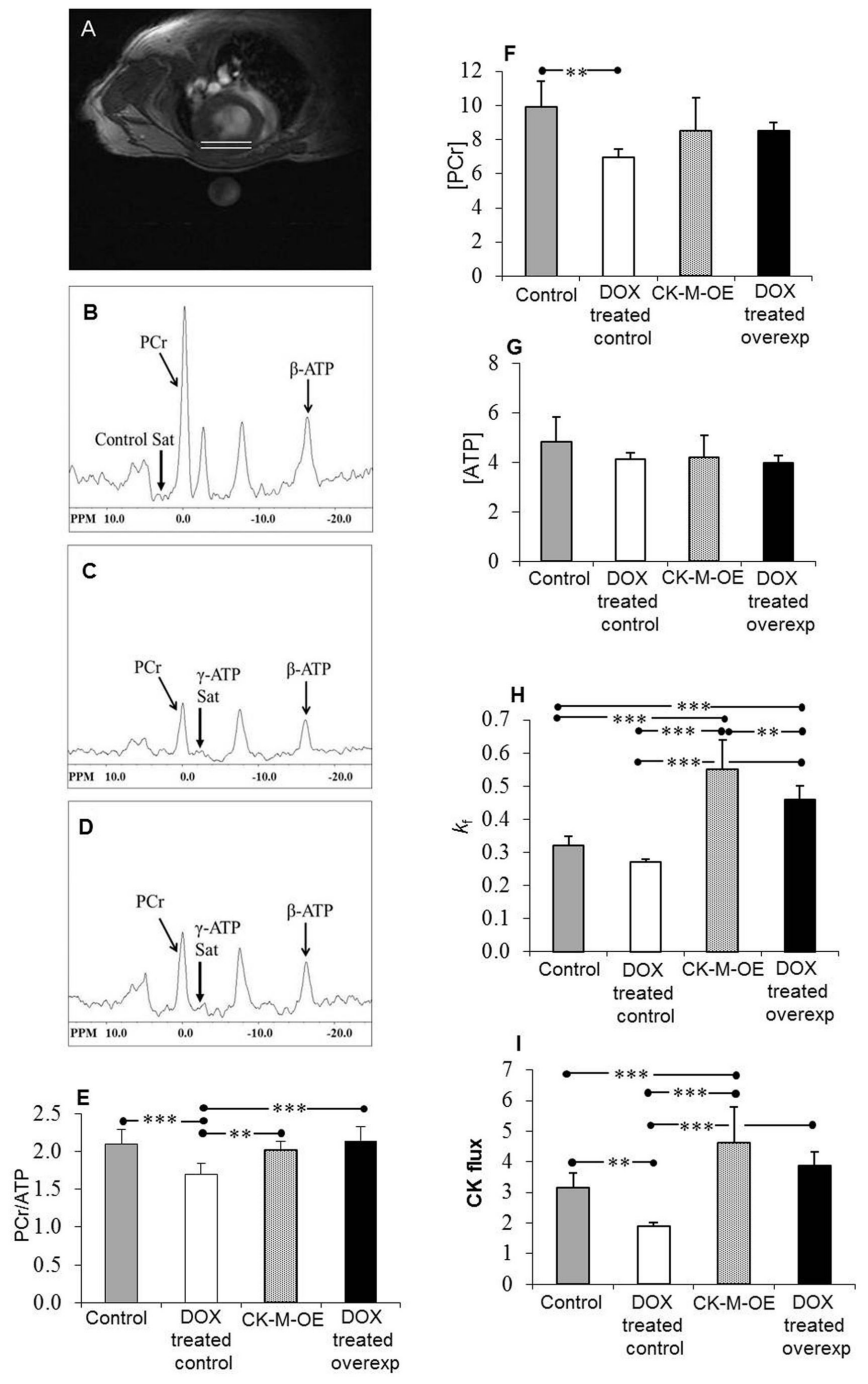
*In vivo* measures of myocardial energetics in DOX administered mouse hearts were obtained with noninvasive ^31^P MRS. (**A**) Typical transverse ^1^H MR image of a mouse at the mid-left ventricle withthe location of ^31^P MR cardiac voxel denoted between the white lines. (**B**) ^31^P MR spectrum with control saturation and TR=10s and NEX=16. (**C**) Spectrum with γ-phosphate of ATP saturated with TR = 6 s, NEX = 32. (**D**) Spectrum with γ-phosphate of ATPsaturation and TR=1.5s, NEX=96. β-ATP; β-phosphate of ATP. (**E**–**I**) Summary of *in* *vivo* energetics (mean+SD) for control (gray bars, *n* = 11), control with DOX (whitebars, *n* = 5), CK-M-OE (bars with dotted background, *n* = 8), and CK-M-OE with DOX (black bars, *n* = 5) mice. (**E**) Cardiac PCr/ATP. (**F**) PCr concentration (µmol/g wet weight). (**G**) ATP concentration (µmol/g wet weight). (**H**) CK pseudo-first-order rate constant (*k*_*f*_, s^-1^). (**I**) ATP synthesis rate through CK (CK flux, μmol/g/s). **P < 0.01, ***P < 0.001.

Representative cardiac MR images and *in vivo* ^31^P MR spectra are shown in Figures 2 and 3. The images and spectra were generally of a high-quality, as previously reported *in vivo* for mice, and showed that at baseline, CK-M-OE had no effect on LV systolic or diastolic volumes, ejection fraction (EF), mass (Table 1), or cardiac high-energy phosphate levels (Figure 3), but it did increase the rate of ATP synthesis through CK (CK flux). These observations are consistent with the original description of these conditional CK-M overexpressing mice [28].

**Table 1:** LV morphology and function by MRI in DOX-treated control and CK-M-OE mice.

		HR, bpm	EDV, µl	ESV, µl	SV, µl	EF, %	CO, ml/min	LVmass, mg
Control	(n=6)	418±20	65.4±8.8	22.0±4	43.4±4	66.5±2	18.1±1	104±8
Control DOX (6wk)	(n=10)	432±46	72.9±13	29.4±8	43.4±5	60.1±4	18.7±3	100±17
Control DOX (8wk)	(n=8)	435±47	72.0±19	36.1±16	35.9±3^b^	51.7±7^a,c,d,e^	15.6±2	101±18
CK-M-OE	(n=6)	440±20	69.1±8.8	23.2±4.6	46.0±4	66.7±3	20.2±1	105±3
CK-M-OE DOX (6wk)	(n=7)	454±56	66.5±10	25.7±7	40.8±4	61.8±5	18.5±2	98±10
CK-M-OE DOX (8wk)	(n=7)	473±12	63.0±13	24.1±6	38.9±7	61.8±3	18.3±3	103±18

Values are means ±SD;*n*, number of mice. HR, heart rate; EDV, end-diastolic volume; ESV, end-systolic volume; SV, stroke volume; EF, ejection fraction;CO, cardiac output, LV, left ventricular.

^a^ p<0.001 compared to control

^b^ p<0.05, ^c^ p<0.01 compared to DOX treated control (6wk)

^d^ p<0.01 compared to DOX treated CK-M-OE (6wk),

^e^ p<0.01 compared to DOX treated CK-M-OE (8wk)

Six weeks after starting DOX, LV volumes, EF and mass were unchanged in control and CK-M-OE mice (Table 1). DOX impaired *in vivo* cardiac energetics as documented by ^31^P MRS which revealed a significant *in vivo* reduction in cardiac PCr/ATP ratio, [PCr], and CK flux in control mice seven weeks after starting DOX, as compared to baseline values (Figure 3). In contrast, CK-M-OE prevented the DOX-induced decline in the *in vivo* cardiac PCr/ATP ratio, [PCr] and CK flux observed in control DOX mice (Figure 3).

DOX-associated contractile dysfunction was first detected at 8 weeks in control mice (Table 1). Mean EF and stroke volume (SV) were modestly but significantly lower in control DOX hearts than at baseline, while mean cardiac output (CO) trended lower and end systolic volume (ESV) trended non-significantly higher (Figure 2 and Table 1). In contrast in CK-M-OE hearts 8 weeks after starting DOX, EF was unchanged from baseline and significantly higher (62±3%) than that in control DOX hearts at the same time (52±7%, p<0.001). In addition, ESV trended lower and CO trended higher in CK-M-OE DOX hearts as compared to control DOX-administered hearts (Table 1).

An increase in the lung/body weight ratio in animal models of HF is often used to assess pulmonary congestion. Although the systolic dysfunction was relatively mild, it is interesting to note that the lung/body weight ratios were significantly increased in control mice following DOX as compared to those treated with saline (*p*<0.001, Table 2) but were not different in CK-M-OE mice following DOX as compared to CK-M-OE administered saline (*p*=0.12, Table 2). Nevertheless, the lung/body weight ratios were similar in control and CK-M-OE mice. In summary, DOX depressed cardiac high-energy phosphates, CK flux and left ventricular EF in control hearts as compared to baseline (Table 1). CK-M-OE improved depressed energetics in DOX hearts and this was associated with significantly better EF and trending better CO and ESV at 8 weeks, along with an attenuation of the development of pulmonary congestion.

**Table 2:** Lung/body weight ratios: lung (mg)/body weight (g) at 8 weeks are shown for control and CK-M-OE mice.

	**Saline**	**DOX**
**Control**	3.6±0.6	5.0±0.3*
**CK-M-OE**	4.4±1.0	5.2±0.6

Mean±SD, n=6-10 for each group *p<0.001 vs control

**Figure 4:**
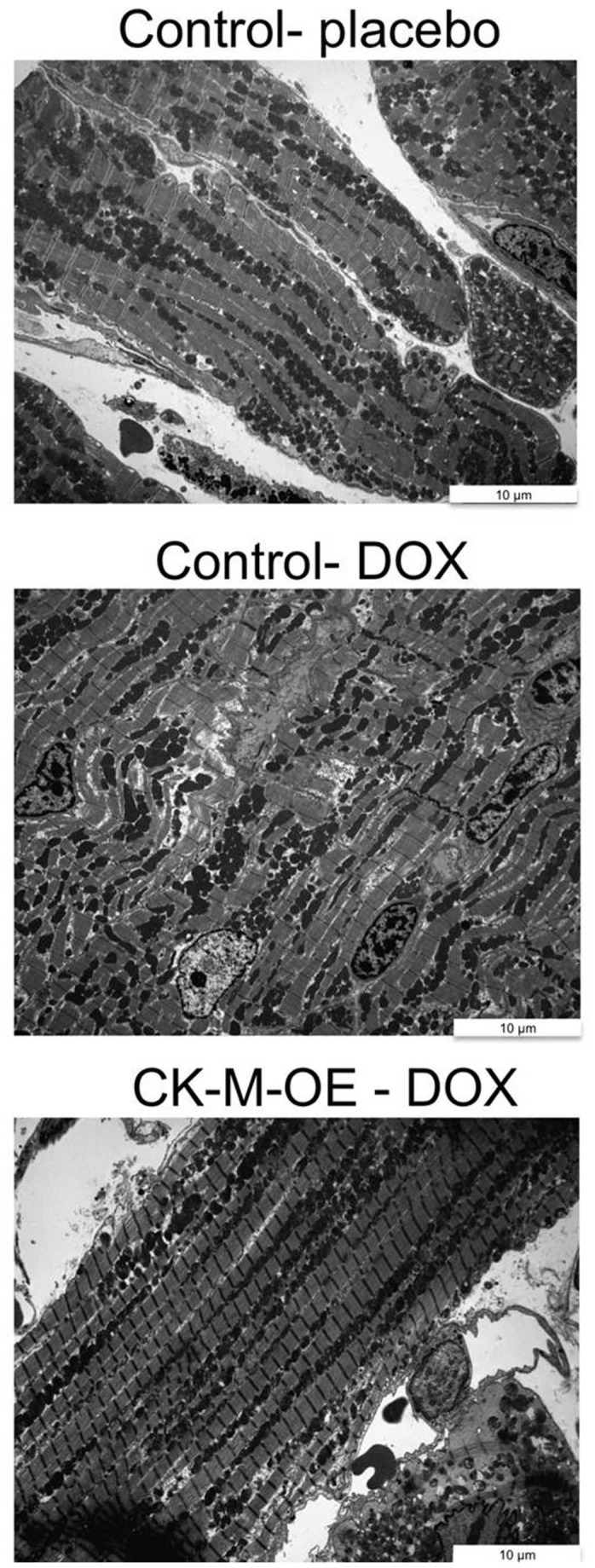
Mild ultrastructural changes are observed in this DOX model. Electron micrograph obtained 8 weeks after randomization to placebo (top panel) or to DOXfrom control (middle panel) and CK-M-OE (bottom panel) indicating mild, patchy myofibrillar loss in DOX hearts.

To determine whether these early metabolic and functional changes were associated with histopathologic changes of DOX cardiotoxicity, electron micrographs were obtained eight weeks after initiation of DOX or placebo. In DOX-administered hearts, there was patchy, very mild vacuolization with minimal myofibrillar loss as compared to saline-treated hearts (Figure 4). However there were no dramatic ultrastructural differences between control DOX and CK-M-OE DOX hearts, and none of the hearts showed severe degenerative changes.

## Discussion

There are several main findings from this *in vivo* study of augmented CK energy metabolism in DOX cardiotoxicity. First, we observe that not only is cardiac PCr/ATP reduced with DOX, as previously reported [35], but that for the first time that the absolute concentration of [PCr] and the *in vivo* rate of ATP synthesis through CK (CK flux) are significantly reduced following chronic DOX administration in control hearts. Second, CK-M overexpression increases the rate of CK flux in placebo-treated hearts but has no effect on PCr/ATP, [PCr] and [ATP] Figure 3) or on contractile function (Table 1), consistent with a prior report [28]. Third, critically, CK-M overexpression prevents the early decline in cardiac high-energy phosphates in DOX mice and attenuates the subsequent contractile dysfunction observed following DOX. Fourth, CK-M overexpression does not appear to alter the early mild ultrastructural changes associated with DOX cardiotoxicity. Fifth, CK-M over-expression increases survival following DOX. These findings demonstrate the DOX-induced depression of cardiac CK-related energy metabolism contributes, at least in part, to the early contractile dysfunction and reduced survival with DOX cardiotoxicity in this model.

Although DOX is an extremely effective antineoplastic drug, the cumulative dose of DOX that can be administered is often limited by cardiotoxicity [1,2]. A number of potential mechanisms of DOX cardiotoxicity have been identified [36-38] but no accepted, evidence-based means to prevent DOX cardiotoxicity is currently available [39]. Because of the prior evidence pointing to DOX-induced mitochondrial and energetic abnormalities, the focus of this work was on the effect of augmented CK energy metabolism on contractile function and survival following DOX.

DOX consistently reduces myocardial high-energy phosphates and energy metabolism across models and species. In isolated myocytes, DOX reduces high-energy phosphates by 30% or more in many studies [2]. In isolated perfused hearts DOX lowers cardiac PCr and ATP acutely [22,40] and, in hearts previously administered DOX on a chronic basis, the high-energy phosphate reductions were even larger [19]. *In vivo* cardiac PCr/ATP declines after chronic DOX administration and the decline precedes contractile abnormalities and predicts eventual mechanical dysfunction [27]. The current *in vivo* findings extend those prior observations of an early decline of *in vivo* cardiac PCr/ATP and demonstrate here that CK ATP kinetics (both CK k_f_ and CK flux) are also reduced early after DOX and are not merely a late consequence in end-stage cardiotoxicity.

There are several mechanisms that likely contribute to the altered cardiac energetics in DOX cardiotoxicity. Oxidative stress (OS) is the most commonly described mechanism for DOX induced cardiotoxicity [41-44] and this contributes to mitochondrial swelling, apoptosis and eventual myofibrillar loss. Myocellular loss with fibrotic replacement, if present, could potentially explain some high-energy phosphate loss (measured as µmol/g tissue) observed here, although we did not observe significant degenerative loss or fibrosis (Figure 4). Mitochondrial dysfunction and uncoupling induced by DOX along with direct effects on the energy sensor AMP-activated protein kinase (AMPK) [45], could also limit ATP production along with DOX-impairment of fatty acid and glucose metabolism [1]. The DOX-induced reduction in CK flux is likely not due to myocyte loss alone since the former (~50%) is out of proportion to the latter and ATP levels are normal. OS and DOX, individually and together, have been shown to impair the structure and function of both the myofibrillar and mitochondrial isoforms of CK [2,46-48]. Oxidative CK injury is further exacerbated in the presence of ferrous iron [49]. A sub-fraction of cytosolic CK enzymes are associated with ATP utilizing sites (actomyosin ATPase and SR Ca^2+^ATPase) and the peroxynitrite-related protein nitration that occurs in DOX cardiotoxicity specifically interferes with myofibrillar CK-M [26,50]. The mitochondrial CK isoform is also susceptible to DOX-induced OS injury with resulting dimerization, reduced activity, impaired binding to cardiolipin and reduced channeling of high-energy phosphates from the mitochondria to the cystosol [1,51]. Thus several well-established factors likely contribute to cardiac energetic abnormalities that occur relatively soon after DOX administration. Cardiac-specific conditional CK-M overexpression was able to augment the reduced cardiac CK flux in DOX hearts back to CK ATP synthesis rates of control, saline-treated hearts (Figure 3). Antioxidant and other approaches designed to limit OS and CK injury following DOX would have been reasonable alternative approaches [52], but the “brute-force” CK-M overexpression strategy used here was specific for CK-M and effective in normalizing myofibrillar CK ATP delivery rates in DOX hearts.

CK-M overexpression normalized cardiac CK flux and [PCr] in DOX hearts and improved their depressed mean left ventricular ejection fractions, suggesting that the early contractile dysfunction in DOX-cardiotoxicity is related, at least in part, to insufficient myofibrillar energy delivery. In a prior study of TAC-induced left ventricular hypertrophy and heart failure, CK-M overexpression increased cardiac high-energy phosphates and attenuated the depressed contractile function resulting from TAC [28]. The contractile benefit resolved when the CK-M overexpression was withdrawn by manipulation of the CK-M transgene while in animals in which CK-M overexpression was maintained survival was significantly better after TAC as compare to littermates. Those results suggest that inadequate energy supply underlies, at least in part, contractile dysfunction in heart failure [28]. The current findings of improved contractile function and survival after DOX are consistent with the prior report in TAC and indicate in at least two murine models of contractile dysfunction, TAC-induced heart failure and DOX-cardiotoxicity, that inadequate energy supply contributes to mechanical dysfunction or that the dysfunctional, failing heart is energy starved as it relates to CK metabolism.

A potential criticism of this study is that the focus was on the early contractile dysfunction of DOX-cardiotoxicity rather than on end-stage heart failure. The early time was chosen for two reasons. First, contractile function is followed clinically in DOX-treated patients such that if an early decline in ejection fraction is noted, DOX dosing can be held or limited in the hope of avoiding irreversible injury and subsequent heart failure. Thus the early dysfunction has some clinical relevance. Second, late or end-stage DOX cardiotoxicity is associated with irreversible myocellular and myofibrillar loss. Augmentation of metabolic energy reserve in surviving myocytes is unlikely to affect function following irreversible injury with apotosis and necrosis. Thus the early stages of DOX cardiotoxicity offered a clinically based model, unlike myocardial infarction with necrosis, to evaluate the role of energy starvation in dysfunctional or failing hearts without dense necrosis and cellular loss. Because of the negative impact of DOX on mitochondrial CK, it would be of interest to determine whether over-expression of the mitochondrial CK isoform would also improve contractile dysfunction.

In summary, CK-M-OE increases depressed cardiac high-energy phosphate levels and rates of ATP synthesis through CK, the primary energy reserve reaction, following DOX administration and this is associated with a significant improvement in left ventricular EF and in survival in DOX animals. These findings indicate that the contractile dysfunction occurring early after chronic DOX administration is related, at least in part, to inadequate myofibrillar energy supply and suggest that strategies that improve myocardial metabolism or limit energetic decline may provide an important new avenue in limiting the consequences of DOX-cardiotoxicity and thereby extending its therapeutic impact.
